# Development of an assessment technique for basic science retention using the NBME subject exam data

**DOI:** 10.1186/s12909-022-03842-5

**Published:** 2022-11-09

**Authors:** Alexandra R. Matus, Lyndsey N. Matus, Adam Hiltz, Tian Chen, Bhavneep Kaur, Pamela Brewster, Zhen Sun, Deepa Mukundan, Lori M. DeShetler, Jeremy J. Laukka, Bindu Menon

**Affiliations:** 1 Department of Medical Education, The University of Toledo College of Medicine and Life Sciences, Toledo, OH USA; 2 Department of Mathematics and Statistics, The University of Toledo, Toledo, OH USA; 3 Department of Medicine, The University of Toledo College of Medicine and Life Sciences, Toledo, OH USA; 4 Department of Pediatrics, The University of Toledo College of Medicine and Life Sciences, Toledo, OH USA; 5 Departments of Medical Education and Neurology, The University of Toledo College of Medicine and Life Sciences, 318 Mulford Library, Mail Stop 1050; 3000 Arlington Ave, Toledo, OH USA; 6 Departments of Medical Education and Physiology and Pharmacology, The University of Toledo College of Medicine and Life Sciences, 3105B CCEB; 2920 Transverse Dr, Toledo, OH USA

**Keywords:** Undergraduate Medical Education (UME), Basic science knowledge retention, Foundational sciences, Subject exam, Quantitative analysis of assessments

## Abstract

**Introduction:**

One of the challenges in medical education is effectively assessing basic science knowledge retention. National Board of Medical Examiners (NBME) clerkship subject exam performance is reflective of the basic science knowledge accrued during preclinical education. The aim of this study was to determine if students’ retention of basic science knowledge during the clerkship years can be analyzed using a cognitive diagnostic assessment (CDA) of the NBME subject exam data.

**Methods:**

We acquired a customized NBME item analysis report of our institution’s pediatric clerkship subject exams for the period of 2017–2020 and developed a question-by-content Q-matrix by identifying skills necessary to master content. As a pilot study, students’ content mastery in 12 major basic science content areas was analyzed using a CDA model called DINA (deterministic input, noisy “and” gate).

**Results:**

The results allowed us to identify strong and weak basic science content areas for students in the pediatric clerkship. For example: “Reproductive systems” and “Skin and subcutaneous tissue” showed a student mastery of 83.8 ± 2.2% and 60.7 ± 3.2%, respectively.

**Conclusions:**

Our pilot study demonstrates how this new technique can be applicable in quantitatively measuring students’ basic science knowledge retention during any clerkship. Combined data from all the clerkships will allow comparisons of specific content areas and identification of individual variations between different clerkships. In addition, the same technique can be used to analyze internal assessments thereby creating an opportunity for the longitudinal tracking of student performances. Detailed analyses like this can guide specific curricular changes and drive continuous quality improvement in the undergraduate medical school curriculum.

## Introduction

The longitudinal emphasis and enduring value of basic science education in medical school continue to be a focus for basic and clinical science educators who strive to develop a horizontally and vertically integrated curriculum. Since the Flexner report of 1910 [1], which shaped the standards of medical education, students' expectations have transcended what has been established as precedence over a century ago. The voice of dissatisfaction from students over the lack of relevance and application of basic science education during their clinical education has become louder each year. In parallel, faculty have complained about students’ repeated failure to recall relevant basic science at the bedside, operating room, and outpatient clinical environments. The education, experiences, and natural evolutionary changes in the practice of medicine have continued to expand since 1910. A core value that helps define the importance of dynamic change in medical education is the school's continuous quality improvement (CQI) initiative that uncovers, collects, and interprets outcomes that can influence and guide major/minor curricular reforms. Along the journey to raise the bar of basic science education, an important ingredient is the identification of tools and resources to help unite and remove barriers that define the 2 + 2 model of 1910 and redefine a protensive story that begins on the first day of medical school. One element of our institution’s CQI process and the core aim of this paper was to leverage the itemized student data from the National Board of Medical Examiners (NBME) subject examinations of our clerkships as another tool to advance basic science integration and evaluate and promote retention and retrieval practices.

In basic science disciplines such as biochemistry, studies have reported that less than 50% of knowledge is retained when learning activities occur without associated clinical correlations in the preclinical years [2]. The literature suggests the need to recalibrate and redefine the value of retrieval, spacing, and interleaving to reinforce core material that has an enduring purpose across the milestones that shape a career in medicine. Studies have shown that integration improves diagnostic accuracy and understanding of key clinical features [3]. However, many medical schools in the U.S. find it challenging to determine the best way to integrate information so that students can make lasting connections [4]. Significant issues that challenge the latitude in basic science curriculum redesign include the competing priorities of an earlier clinical experience, a robust health science education, professional/leadership development and opportunities, professional identity formation/wellness programs, and specialized pathways/tracks of distinction, to name a few. The objective of shaping future physicians is grounded in transferable and acquired skills that define a culture of educational excellence. This premise of achieving excellence must include innovative practices that complement the value and importance of basic science competence and identify tools that will help achieve that very goal.

U.S. medical schools variably use NBME subject examinations as a summative end-of-clerkship assessment of learning outcomes. In fourth-year medical students, studies have shown important correlations between NBME subject exams and in-house exam performance outcomes that assess the application of medical knowledge, skills, and clinical reasoning, which are essential for providing patient care under supervision [5–7]. However, we did not find many studies that quantitatively measured basic science knowledge acquisition using subject exam data. We hypothesized that quantitative measurement in retention of basic science knowledge in the clerkship NBME subject exam can help inform our CQI process and guide decisions that will correct deficiencies in the foundational science curriculum. The objective of this study is two-fold: 1) identifying new opportunities to challenge our pedagogical methods and assumptions about basic science integration and current practices across the continuum of medical education, and 2) measuring comprehension, retention, and retrieval of basic science knowledge that is aligned to their clinical clerkship experience.

## Methods

### Customized NBME subject exam data

A retrospective analysis of the University of Toledo College of Medicine and Life Sciences (UTCOMLS) NBME subject exam item-level data for the pediatrics clerkship during the period of 2017–2020 was conducted to study the effect of pre-clinical curriculum on the retention of basic sciences during clinical years. The study involved data from the academic years of 2017–2018, 2018–2019, and 2019–2020 with about 185 students in each academic year.

**Fig. 1:**
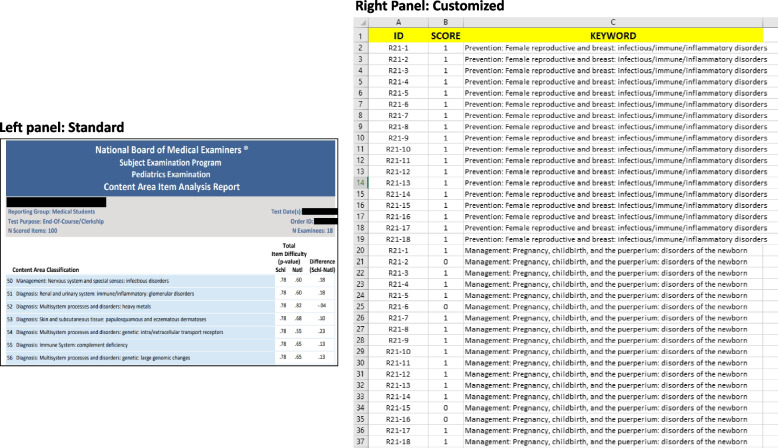
NBME Item analysis reports. Left panel: An excerpt of the standard item analysis report. The screenshot provides information about the item difficulty of each question (item) that appears on an NBME subject exam for the school vs. national. P value indicated probability values of getting the correct answer. For example, question number 50 on this exam was on “Management: Nervous system and special senses: infectious disorders” and our students had a p value of 0.78 compared to a national average of 0.60. Right panel: An excerpt of the customized item analysis report. The Excel sheet provides information on the performance of students in addition to the content area of the question. For example, the above image shows the performances of 18 students (represented in column A as R21-1, R21-2, R21-3, etc.) who rotated in the pediatric clerkship together and took the same exam. Their performances are given in a binary format in column B (SCORE), where 0 indicates wrong answer and 1 indicates correct answer. The excerpt of the report presented in this figure shows the performances of these 18 students on two questions (out of 100 total questions on the exam), which are represented in column C by the KEYWORDs “Prevention: Female reproductive and breast: infectious/immune/inflammatory disorders” and “Management: Pregnancy/childbirth, and the puerperium: disorders of the newborn.” The student’s name is deidentified and an alternate ID is given (see column A; ID). In both cases, the details of the question are not given; rather, the content areas that are tested through this question are provided

NBME subject exams are developed to assess clinically oriented skills and the corresponding foundational science knowledge within the realm of a given clerkship. These exams are often used by medical schools as a portion of their grade for a given clinical rotation. Normally, NBME does not provide details about the questions that appear on subject exams to individual institutions. The standard item analysis report that they provide after each subject exam, a redacted portion of which is shown in Fig. 1 (left panel), only gives the content areas corresponding to each question in the exam, and the “probability value” (p-value) of the institution for that question compared to the national average. This p-value merely provides an estimate of item difficulty with regard to a content area, which is not sufficient information for any kind of quantitative analysis. We obtained approval from the Institutional Review Board and acquired a customized report from NBME that had individual student performance data. A representative portion of the customized item analysis report is shown in Fig. 1 (right panel). Similar to the standard item analysis report, the customized report also contained the keywords representing the content areas assessed for each question (Column C “KEYWORD”; Fig. 1, right panel) that appeared in the subject exam. Each subject exam usually has 100 questions (denoted as assessment items). Thus, the performance of each student across these 100 items was provided in a binary format, where “1” indicated a correct response given by the student, and “0” indicated an incorrect response (Column B “SCORE”; Fig. 1, right panel). Cumulatively, the report contained combined data of 185 students at UTCOMLS across several blocks, with each block having 14–20 students who rotated together (4.5–6 weeks) and took the same exam at the end of the rotation.

### DINA model analysis

We used a cognitive diagnostic assessment (CDA) method to assess skill mastery of students longitudinally and comparatively. CDA measures the strengths and weaknesses of a knowledge domain in terms of the information that is learned and that which has yet to be acquired [8–10]. For this pilot study, we analyzed content mastery in 12 key basic science content areas using the deterministic input, noisy “and” gate (DINA) model [11]. The DINA model is a type of CDA that predicts the probability of mastery of latent variables such as skills or attributes [12]. Using the DINA model to assess skill mastery among students allowed us to overcome traditional limitations of item analysis, which include difficulty in isolating which skill deficits are responsible for an incorrect response when a question assesses multiple skills at once. For example, if an item analysis were conducted for an exam, and it only looked at the percentage correct for questions involving “skill A,” it would likely underestimate the student’s proficiency in “skill A” due to confounding by the other skills required for the questions they had missed. Instead, the DINA model utilizes an expectation–maximization algorithm to estimate the most likely value of parameters in a statistical model; in the case of this study, the algorithm thus predicts the most likely explanation of mastery or non-mastery of skills for a student to explain their examination performance [13]. Compared to the deterministic input, noisy “or” gate model (DINO), DINA is non-compensatory, meaning that lack of mastery of a skill cannot be rescued by mastery of other skills required by a given item. Due to the complexity of clinical presentations, we assumed that mastery of all skills tested by a given question required a correct response by the student. All instances of a student answering a question without mastery of all required skills were considered to be correct by chance (i.e., guessing). To identify which skills were required for each question, a Q-matrix had to first be developed [14].

### Development of Q-matrix

A Q-matrix [14] is a confirmatory matrix that identifies the skills required to answer each item in an assessment in a binary format, where “1” indicates the requirement of a skill to answer the item, and “0” indicates that a skill is not required by an item. We developed an $I\times J$ Q-matrix, where *j* different skills or attributes were required to correctly answer *i* questions from the NBME Pediatrics Subject Exam. For example, to correctly answer question *i* on “Diagnosis: Gastrointestinal (GI) system: congenital disorders,” we determined that students needed to possess knowledge (skill) in 3 content areas: *j*_*1*_, diagnostic principles; *j*_*2*_, GI system; and *j*_*3*_, genetics. A total of 149 skills were identified from the entire report. A partial list of skills identified is shown in Table 1. Each question had 3–5 skills matched to them. Questions that assessed the same combination of skills sometimes appeared on the same exam or across different exams. For organizational purposes, these were assigned the same numerical identifier but with an additional, unique alphabetical classifier to differentiate between different questions assessing the same skills. A sample list of questions mapped to the corresponding skills (Q-matrix) is shown in Table 2. We used 12 major content areas (major organ systems such as cardiovascular, respiratory, neuromuscular, etc.) for our pilot study, and the results are provided in this manuscript.

**Table 1:** Partial list of skills used for DINA model analysis

Skills	Name
SK-1	**Diagnosis**
SK-2	**Management**
SK-3	**Prevention**
SK-4	**Foundation**
SK-5	**General principles**
SK-6	**Pediatric**
SK-7	**Pregnancy/childbirth/puerperium**
SK-8	**Organ systems**
SK-9	**Multisystem Processes and Disorders**
SK-10	**Cardiovascular system**
SK-11	**Respiratory system**
SK-12	**Renal and urinary system**
SK-13	**Gastrointestinal system**
SK-14	**Endocrine system**
SK-15	**Male reproductive system**
SK-16	**Female reproductive system**
SK-17	**Nervous system and special senses**
SK-18	**Musculoskeletal system**
SK-19	**Blood and lymphoreticular system**
SK-20	**Skin and subcutaneous tissue**
SK-21	**Behavioral health**
SK-22	**Social sciences**
SK-23	**Immune system**
SK-24	**Congenital disorders**
SK-25	**Inflammatory disorders**

The knowledge in each of the content areas assessed in the NBME subject exams are marked as a “skill” necessary to answer the assessment items. A representative list of the major skills (30 out of 149 total skills) that appear in the pediatric clerkship subject exams is given above

**Table 2:** Sample Q-matrix used for DINA model analysis

Question IDs	Keywords	Skills
Q1	Prevention: Female reproductive and breast: infectious/immune/inflammatory disorders	SK-3, SK-16, SK-31, SK-27, SK-25, SK-32
Q2	Management: Pregnancy, childbirth, and the puerperium: disorders of the newborn	SK-2, SK-7, SK-30
Q3	Diagnosis: Respiratory system: disorders of the pleura, mediastinum, chest wall	SK-1, SK-11, SK-33
Q4	Diagnosis: Skin and subcutaneous tissue: infestations and nonvenomous bites/stings	SK-1, SK-20, SK-34
Q5	Diagnosis: Pregnancy, childbirth, and the puerperium: obstetric complications	SK-1, SK-7, SK-35
Q6	Diagnosis: Cardiovascular system: congenital disorders	SK-1, SK-10, SK-24
Q7	Diagnosis: Musculoskeletal system: traumatic and mechanical disorders	SK-1, SK-18, SK-29
Q8	Foundation: Skin and subcutaneous tissue: viral infections	SK-4, SK-20, SK-36
Q9	Diagnosis: Musculoskeletal system: immunologic disorders	SK-1, SK-18, SK-32
Q10	Diagnosis: Respiratory system: infectious/immune/inflammatory disorders: upper airways	SK-1, SK-11, SK-27, SK-32, SK-25, SK-37
Q11	Management: Multisystem processes and disorders: shock	SK-2, SK-9, SK-38
Q12	Diagnosis: Nervous system and special senses: congenital disorders	SK-1, SK-17, SK-24
Q13	Prevention: Cardiovascular system: infectious disorders	SK-3, SK-10, SK-27
Q14	Diagnosis: Respiratory system: infectious/immune/inflammatory disorders: lower airways	SK-1, SK-11, SK-27, SK-32, SK-25, SK-39
Q15	Foundation: Endocrine system: pituitary disorders	SK-4, SK-14, SK-40
Q16	Diagnosis: Multisystem processes and disorders: viral infections	SK-1, SK-9, SK-36
Q17	Diagnosis: Multisystem processes and disorders: immunologic and inflammatory disorders	SK-1, SK-9, SK-32, SK-25
Q18	Gen Principle: Social Sciences: death and dying:pain management	SK-5, SK-22, SK-41, SK-42
Q19	Management: Immune System: HIV/AIDS	SK-2, SK-23, SK-43
Q20	Diagnosis: Blood and lymphoreticular system: neoplasms of blood and lymphatic system	SK-1, SK-19, SK-44

A representative sample of the question by skills Q-matrix that was developed for the DINA model analysis is given above. Each question that appears in the exam is mapped to the skills necessary to answer that question

### Comparison of different curricula

In 2017, UTCOMLS implemented a redesigned curriculum named “Rocket Medicine," emphasizing a competency-based curriculum with early clinical experience. There was an enhanced focus on clinical medicine preparation and skills and early clinical experiences compared to the legacy curriculum. Since this major curriculum change happened during the study period, we were able to use the current method to compare the performance of students from the previous legacy curriculum with the Rocket Medicine curriculum. As such, the analysis of content mastery was compiled separately for each block. For example, in the pediatric clerkship cohort that we examined for the year 2017–2018, 9 blocks of exam-takers experienced the legacy curriculum, and 2 blocks of exam-takers received the new Rocket Medicine curriculum. The DINA model was applied for each block separately due to the use of different questions across exams. This provided an estimate of content mastery for each block independent of the other blocks. However, mastery of individual skills was averaged for both the legacy and Rocket Medicine curricula, which was then used to compare the two sets. Due to the variability in the number of students per block or exam, a weighted average of content mastery was calculated. Statistical significance was calculated by regression analysis (R foundation for statistical computing, Vienna, Austria) using parametric and nonparametric analyses. The block-by-block data was tested for normality using the Shapiro–Wilk and Q-Q plot tests. If the distribution was not normally distributed the least squares estimator was used to test for statistical significance.

## Results

**Fig. 2:**
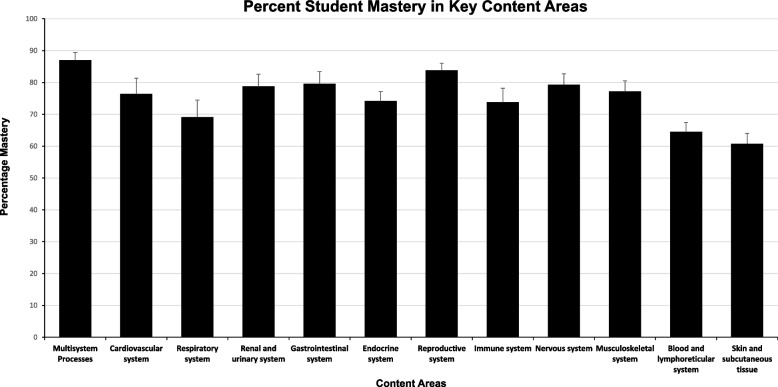
Percent Student Mastery in Key Content Areas. The average percentage mastery of the 2017–2020 cohort of pediatric clerkship students at UTCOMLS in key content areas analyzed in this pilot study are represented in the figure. Error bars represent ± SE

In our pilot study, we analyzed content mastery in select content areas (major organ systems in the body) that are designated as skills (Fig. 2). Some systems that include “Multisystem processes” and “Reproductive system” reported a higher percentage of student mastery (87.01 ± 2.4% and 83.8 ± 2.3%, respectively), whereas “Skin and subcutaneous tissue” and “Blood and lymphoreticular system” (60.7 ± 3% and 64.7 ± 2.9%, respectively) reported lower performance. The outcome of these findings validated the importance of proper retrieval processes and spaced integration of core topics in the basic sciences.

**Fig. 3:**
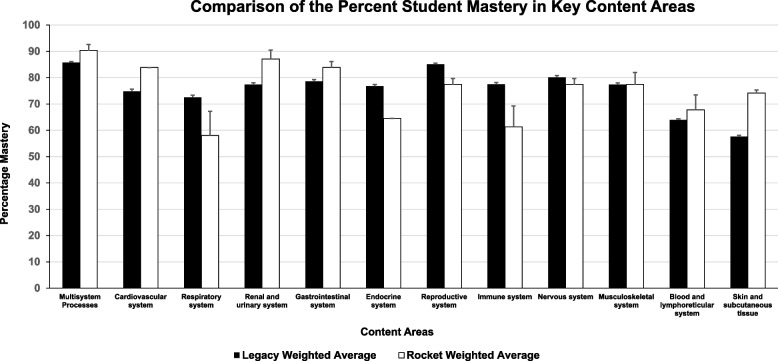
Comparison of the Percent Student Mastery in Key Content Areas. The average percentage mastery of the 2017–2020 cohort of pediatric clerkship students at UTCOMLS analyzed separately based on their foundational science curricula (Rocket Medicine vs. Legacy) are represented in the figure. Error bars represent ± SE

The cohort of students in the pediatric clerkship whose performance we analyzed and interpreted included students from both the legacy and the new Rocket Medicine curriculum. Accordingly, we separated the students based on their curriculum and analyzed them differentially, which allowed us to compare results from both the legacy and the Rocket Medicine curriculum (Fig. 3). Students from the Rocket Medicine cohort, in general, outperformed students from the legacy curriculum in the “Cardiovascular system” (83.87% vs. 74.85%, respectively, p < 0.05) and “Skin and subcutaneous tissue” (74.19% vs. 57%, respectively). With that said, we found that students from the legacy curriculum performed better than students from the Rocket Medicine curriculum in the “Reproductive system,” 85.07% (legacy) mastery versus 77.41% (Rocket Medicine), and “Immune system,” 77.5% (legacy) mastery versus 61.31% (Rocket Medicine).

## Discussion

Our team was able to identify areas where our students demonstrated strength in content and other areas where their performance was weaker from the results of this pilot study using pediatric clerkship data. We found this finding informative as it validated assumptions about space, interleaving, and system alignment/integration of material within the foundational science curriculum of the M1 and M2 academic years. Furthermore, it informs and guides medical school faculty on ways to improve instruction, integration, and assessment of the content where mastery did not meet a standard of competence. In addition, we now have an opportunity to compare the above data from students in their clerkship years with this cohort’s internal assessment scores from their preclinical curriculum. Of note, we have shown that this model can be applied to analyze internal assessments similarly [15]. If analysis of the preclinical assessments shows the same trend (weak performance in a particular content area) as the analysis of clerkship subject exams, instruction can be modified to improve student performance. In contrast, if the internal assessments do not reflect a similar trend, that would indicate the probability that students are not able to retain the information in the clinical years. These foundational science concepts could be reinforced during the clerkship years by meaningful integration with clinical sciences during their clinical rotations. Thus, instead of perceiving student performances as silos during the clinical and preclinical years, this model presents an opportunity to connect the data and track mastery longitudinally using the principles of cognitive science. Another interesting observation was that the systems in the legacy curriculum that performed better were delivered in the M2 year preceding the clerkship experience, suggesting that curriculum timing influences the basic recall of important material. Together, the findings in this pilot suggest that basic science integration, which many of our clerkships foster through our CQI process, facilitates enhanced long-term retention.

Medical education continues to undergo an evolutionary change from the era of Abraham Flexner (1866–1959) [16]. The community of scholars and educators across the landscape of medical schools has imparted innovative programs that contribute to established trends in pursuit of educational excellence [17–19]. Major curriculum changes are challenging and impact all aspects of the curriculum spanning from teaching pedagogy to assessment. Identifying tools like the NBME subject examination can help guide best practices and measure retrieval and retention of basic science knowledge [20]. The outcomes have purpose and meaning in being one source of insightful knowledge to address CQI in applying basic science material in clinical clerkships. In addition to facilitating focused improvements in the foundational science curriculum, this method can be easily modified to assess the learning outcomes of students on in-house assessments. Together with the core clerkship outcomes, these data can be used for tracking longitudinal progress across the continuum of undergraduate medical education. Moreover, using this approach creates feasibility in monitoring the success and CQI of new pedagogies. By analyzing the data over a sustained period, we can evaluate the efficacy of teaching methods and better define how to best develop our faculty as medical educators. As David Kern eloquently articulated in his 6-step approach to curriculum development [21], assessment of student performance in specific areas is a valuable tool to evaluate the effectiveness of curricular changes. The transformative work of Kern supports assessment tools, like the NBME subject exam, to guide curriculum changes creating an environment where learners develop a working memory that allows them to recall and apply in a clinical environment.

This study suggests that the model presented here provides meaningful data only if multiple content areas from one assessment are analyzed. It is not possible to individually determine or extrapolate mastery in one content area by itself. However, for our purposes, interpreting the outcome data for the content areas is a value added to the CQI process and curriculum planning. Although this is a pilot study, limitations include the inability to differentiate if the same content area is repeated in other questions and how the student performance on those questions may or may not have changed. We circumvented this issue by naming the content area differently each time while still mapping it to the same skills.

## Conclusion

This model that we developed will allow us to longitudinally analyze student performances based on a cognitive diagnostic assessment. This is beneficial to the CQI process of the institution in several different ways. For instance, as a next step, we intend to obtain data from all the clerkships and prepare similar reports. This will allow us to examine the cumulative data of various content areas and more importantly compare the individual variations between different clerkships. As an example, it would be advantageous to learn if the performance on “Cardiovascular system” differs between the pediatrics, internal medicine, and family medicine clerkships. In order to achieve this, we will have to compile the content areas and the corresponding list of skills from all the different clerkships. While we expect many of the major content areas to remain the same, additional content areas, if any, will be added to the same document. Detailed analyses like these will help the curriculum leaders to make decisions regarding whether more focus is needed on child versus adult pathologies and corresponding basic science knowledge. Another plan is to analyze the performance of struggling students on internal assessments. If there are recurring patterns, it will allow us to conduct early interventions as well as long-term planning. Thus, we believe that if followed up with further research, this method can potentially evolve into a standardized process for CQI in medical schools across the nation.

## Data Availability

The datasets generated and analyzed are available from the corresponding author on reasonable request.
